# Supplementary material for: HPLC-DAD-qTOF Compositional Analysis of the Phenolic Compounds Present in Crude Tomato Protein Extracts Derived from Food Processing
Source: Molecules. 2021 Oct 23;26(21):6403. doi: 10.3390/molecules26216403 (PMC8587773; doi:10.3390/molecules26216403)
Supplement: Supplementary file 1 [file molecules-26-06403-s001.zip › molecules-1405460-supplementary.pdf]

molecules

Supplementary

Table S1. Retention time (RT), experimental molecular mass (Mr. Exp.), calculated molecular mass (Mr. Calc.) and the differences between the two masses (Diff) present in tomato peels, tomato pomace and tomato protein.

Peak	Compound	Extract*	RT	Mr Exp.	Mr Calc.	Diff (ppm)
**1**	Coumaric acid 1.	**1**<br>**2**	3.4<br>2.3	164.0480	164.0473	4.98
**2**	5-*O*-caffeoylchlorogenic acid	**1**	5.3	354.0967	354.0951	4.57
**3**	Caffeic acid-*O*-hexoside 1	**1**<br>**3**	5.5<br>5.1	342.0959	342.0951	2.5
**4**	Homovanillic acid glucoside	**1**<br>**3**	5.9<br>5.9	344.1119	344.1107	3.3
**5**	Chlorogenic acid	**1**<br>**2**<br>**3**	6.1<br>6.1<br>5.8	354.0955	354.0951	1.28
**6**	Caffeic acid-*O*-hexoside 2	**1**<br>**3**	6.1<br>5.8	342.0960	342.0951	2.56
**7**	Rutin-*O*-hexoside	**1**<br>**3**	6.2<br>6.0	772.2106	772.2062	5.63
**8**	Cryptochlorogenic acid	**1**<br>**3**	6.3<br>6.0	354.0949	354.0951	-0.45
**9**	Naringenin-*C*-diglycoside	**1**<br>**2**	6.4<br>6.5	596.1745	596.1741	0.69
**10**	Coumaric acid glucoside	**1**	6.4	326.1007	326.1002	1.63
**11**	Caffeic acid	**1**<br>**3**	6.6<br>6.3	180.0431	180.0423	4.41
**12**	Ferulic acid glucoside 1	**1**<br>**3**	6.7<br>5.7	356.1132	356.1107	6.91
**13**	Vicenin-2	**3**	6.6	594.1593	594.1585	1.41
**14**	Protocatechuic acid	**1**<br>**2**<br>**3**	6.7<br>6.5<br>6.1	154.0266	154.0266	0.15
**15**	Ferulic acid Glucoside 2	**1**	6.7	355.1056	356.1133	7.11
**16**	Caffeic acid-*O*-hexoside 3	**1**	6.8	342.0967	342.0951	4.86
**17**	Coumaroylquinic acid	**1**	6.9	338.1027	338.1002	7.56
**18**	Coumaric acid 2	**1**<br>**2**<br>**3**	7.2<br>7.6<br>7.3	164.1073	164.0473	-1.49
**19**	Rutin-*O*-pentoside	**1**<br>**2**<br>**3**	7.2<br>7.4<br>7.3	742.1963	742.1956	0.94
**20**	Feruloylquinic acid	**1**	7.3	368.1120	368.1107	3.31
**21**	Naringenin-*C*-glucoside	**2**<br>**3**	7.3<br>7.5	434.1214	434.1213	0.3
**22**	Naringenin-*O*-glucoside 1	**3**	7.6	434.1223	434.1213	2.27
**23**	Rutin	**1**<br>**2**<br>**3**	7.7<br>7.9<br>7.8	610.1539	610.1534	0.8
**24**	Eriodyctyol-*O*-glucoside 1	**3**	7.7	450.1177	450.1162	3.2
**25**	Phloretin-*C*-diglycoside	**2**<br>**3**	7.8<br>7.9	598.1905	598.1898	1.26
**26**	Dicaffeoylquinic acid 1	**1**<br>**2**<br>**3**	7.9<br>8.1<br>7.9	516.125	516.1268	-3.5

**27**	Dicaffeoylquinic acid 2	**1**<br>**2**<br>**3**	8.0<br>8.2<br>8.0	516.1277	516.1268	1.71
**28**	Quercetin-3-galactoside	**1**<br>**3**	8.1<br>7.9	464.0989	464.0955	6.71
**29**	Apigenin-7-*O*-glucoside	**3**	8.3	432.1073	432.1056	3.81
**30**	Dicaffeoylquinic acid 3	**2**	8.4	516.1277	516.1268	1.98
**31**	Kaempferol-3-*O*-rutinoside	**1**<br>**3**	8.5<br>8.4	594.1602	594.1585	2.99
**32**	Eriodityol-*O*-glucoside 2	**3**	8.4	450.1195	450.1162	7.37
**33**	Naringenin-*O*-glucoside 2	**3**	8.5	434.1224	434.1213	2.56
**34**	Naringenin-*O*-glucoside 3	**1**<br>**3**	8.6<br>8.5	434.1251	434.1213	8.67
**35**	Eriodityol-*O*-glucoside 3	**3**	8.7	450.1195	450.1162	7.37
**36**	Eriodictyol	**1**<br>**3**	9.7<br>9.5	288.0639	288.0634	1.83
**37**	Quercetin	**1**<br>**3**	10.0<br>9.9	302.0434	302.0427	2.41
**38**	Naringenin 1	**1**<br>**2**<br>**3**	10.2<br>10.5<br>10.4	272.0686	272.0685	0.34
**39**	Apigenin	**3**	10.7	270.0548	270.0528	7.3
**40**	Naringenin 2	**1**	10.8	272.0688	272.0685	1.21

*Extract: Type of samples; Sample 1: tomato pomace (peel, outer skins and seeds); Sample 2: tomato skins; Sample 3: the tomato protein extract.

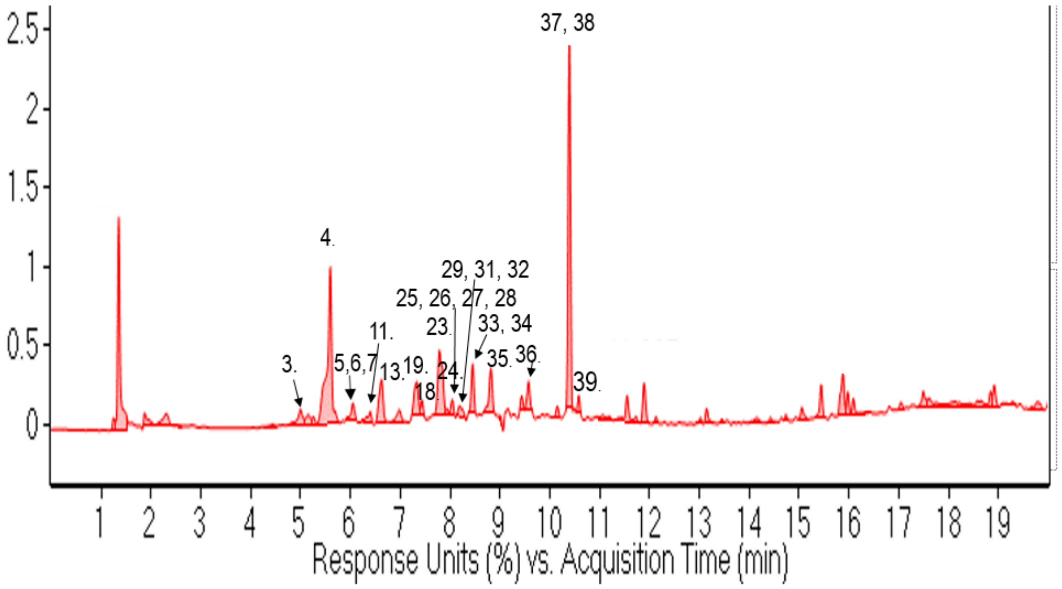

Figure S1. An example of UV chromatogram at 280 nm of a tomato protein extract.

Table S2. Intensities of the main peaks of phenolic compounds that were identified in different tomato extracts by HPLC-DAD-qTOF.

Peak number	Compound	Tomato pomace	Tomato protein	Tomato peels
3	Caffeic acid-O-hexoside I.	129617 ± 9070	4888 ± 342	
4	Homovanillic acid glucoside	54507 ± 3820	7180 ± 503	
5	Chlorogenic acid	82631 ± 5780	39779 ± 2780	18542 ±1300
6	Caffeic acid-O-hexoside II.	96959 ± 6790	6183 ± 433	
7	Rutin-O-hexoside	12329 ± 863	6176 ± 432	
8	Cryptochlorogenic acid	149938 ± 10500	5119 ± 358	
11	Caffeic acid	153498 ± 10740	32311 ± 2262	
12	Ferulic acid glucoside I.	4784 ± 335	13827 ± 968	
13	Vicenin-2		17864 ± 1250	
14	Protocatechuic acid	20509 ± 1440	172963 ± 12110	35073 ± 2460
18	Coumaric acid II.	124482 ± 8710	23848 ± 1670	8102 ± 567
19	Rutin-O-pentoside	235464 ± 16480	112071 ± 7845	8737 ± 612
23	Rutin	119622 ± 8370	111000 ± 7770	73902 ± 5170
34	Naringenin-O-glucoside III.	21895 ± 1533	21516 ± 1506	
36	Eriodictyol	72970 ± 5108	125440 ± 8781	
37	Quercetin	52211 ± 3655	134669 ± 9430	
38	Naringenin I.	97303 ± 6811	50041 ± 3500	80618 ± 5640
